# Extracorporeal kidney support in neonates and infants: therapeutic perspectives and outcomes in multiple organ dysfunction syndrome—a structured narrative review

**DOI:** 10.3389/fped.2026.1739965

**Published:** 2026-10-09

**Authors:** Gulmira Zhauarova, Dinagul Baesheva, Gulnur Nadirbekova, Kamilla Rakhimova, Bolat Bekishev, Talgat Ibrayev

**Affiliations:** 1 Astana Medical University, Astana, Kazakhstan; 2 University Medical Center Corporate Fund, Astana, Kazakhstan

**Keywords:** acute kidney injury, CKRT, extracorporeal blood detoxification, infants, neonates, sepsis

## Abstract

**Background:**

Multiple organ dysfunction syndrome (MODS) remains a major challenge in the intensive care of neonates and infants and is associated with substantial morbidity and mortality, particularly in the presence of sepsis and acute kidney injury. Conventional kidney replacement and detoxification strategies may be difficult to implement in neonates and low-weight infants because of technical and physiological limitations. Hemodiafiltration (HDF), which combines convective and diffusive clearance mechanisms, has emerged as a potential extracorporeal support modality for metabolic and inflammatory control while maintaining hemodynamic stability.

**Objective:**

To provide a structured narrative review of the available literature on the clinical applications, potential benefits, safety considerations, and technical aspects of hemodiafiltration in neonates and infants with multiple organ dysfunction syndrome.

**Methods:**

This study was conducted as a structured narrative review with systematic search elements. PubMed, Scopus, Web of Science, and the Cochrane Library were searched for publications from January 2018 to October 2025. Earlier landmark publications identified through reference-list screening were additionally included to provide historical and technical context. A structured search and descriptive selection process was used to identify publications relevant to extracorporeal blood purification in neonates and infants. Eligible populations included neonates (<28 days of life) and infants beyond the neonatal period (28 days to <12 months) weighing ≤8 kg with MODS, sepsis, hyperammonemia, or acute kidney injury. Observational studies, case series, registry reports, and review articles addressing HDF or continuous kidney replacement therapy (CKRT) were considered. Studies involving broader pediatric populations were included only when relevant data for eligible neonatal or infant subgroups were separately available. Publications lacking clinically relevant information were excluded from the descriptive synthesis. Owing to substantial heterogeneity in study designs, patient populations, and reported outcomes, findings were synthesized descriptively.

**Results:**

The reviewed literature suggests that HDF may be associated with metabolic stabilization, correction of acid–base disturbances, and improvement in electrolyte balance in selected neonates and infants. Limited case-based evidence has also described hemodynamic stabilization and reduced vasopressor support following extracorporeal therapy; however, these observations remain insufficient to establish a causal treatment effect. Clinical outcomes varied substantially across studies because of differences in patient characteristics, clinical indications, and treatment protocols. Reported complications included hypotension, circuit clotting, and coagulation abnormalities, although their frequency and clinical significance differed considerably among publications.

**Conclusions:**

Current evidence suggests that hemodiafiltration may represent a promising extracorporeal support strategy for selected neonates and infants with MODS, particularly for metabolic stabilization and organ support. However, the available evidence remains limited, heterogeneous, and predominantly based on observational studies and case series. Further multicenter prospective studies are required to clarify optimal indications, treatment protocols, and long-term outcomes.

## Introduction

Multiple organ dysfunction syndrome (MODS) remains a major cause of morbidity and mortality among critically ill neonates and infants, particularly those with congenital heart defects, severe infections, postoperative complications, and acute kidney injury (AKI) (1–4). The development of MODS in neonatal and pediatric intensive care settings is associated with systemic inflammatory activation, metabolic dysregulation, impaired tissue perfusion, and progressive organ dysfunction, frequently resulting in prolonged intensive care support and unfavorable clinical outcomes (5–8).

Extracorporeal blood purification techniques have become an important supportive strategy in critically ill neonates and infants with severe metabolic disturbances, fluid overload, sepsis-associated complications, and renal dysfunction (9–12). Conventional extracorporeal modalities used in neonatal and pediatric intensive care include hemofiltration (HF), hemodialysis (HD), and continuous kidney replacement therapy (CKRT) (13–16).

Hemodiafiltration (HDF) combines convective and diffusive clearance mechanisms, allowing the removal of both small- and middle-molecular-weight solutes, including inflammatory mediators and metabolic byproducts (17–20). Because of these characteristics, HDF has been increasingly explored as a potential extracorporeal support modality in neonates and infants with systemic inflammatory response and MODS (21–24).

The application of HDF in neonates and infants remains technically challenging because of their small body size and age-specific physiological and anatomical characteristics, including low circulating blood volume, vascular access difficulties, and the limited availability of miniaturized extracorporeal systems (25–27). However, recent advances in technologies suitable for neonates and low-weight infants, including CARPEDIEM™, NIDUS, and modified Prismaflex HF20 systems, together with improvements in anticoagulation strategies, have increased the feasibility of extracorporeal therapies in these populations (28–33).

Published clinical studies suggest that HDF may be associated with metabolic stabilization, correction of acid–base imbalance, reduction in lactate levels, and improvement in hemodynamic parameters in selected neonates and infants (13, 22, 34–37). Nevertheless, the currently available evidence remains limited and heterogeneous. Most reports are based on observational studies, registry data, and small case series with variable indications, patient populations, and treatment protocols, limiting direct comparison of findings and the development of standardized recommendations (38–42).

Therefore, this structured narrative review aims to provide a descriptive overview and critical appraisal of the available literature on hemodiafiltration in neonates and infants, with particular attention to low-weight patients weighing ≤8 kg. Particular emphasis is placed on clinical applications, technical considerations, safety aspects, reported outcomes, and areas requiring further investigation. The review seeks to summarize current knowledge while acknowledging the limitations and heterogeneity of the existing evidence base (38).

## Methods

### Review design

This study was conducted as a structured narrative review incorporating systematic literature search elements. The review was designed to provide a descriptive overview of the current literature on hemodiafiltration (HDF) and related continuous kidney replacement therapy (CKRT) modalities in neonatal intensive care settings.

A narrative review approach was selected because the available evidence consists predominantly of observational studies, registry analyses, retrospective cohorts, and case series with substantial clinical and methodological heterogeneity. Therefore, quantitative synthesis and meta-analysis were not considered appropriate.

This review does not represent a formal systematic review and was not intended to generate pooled effect estimates.

### PICOS framework

The review question was structured according to the PICOS framework to define the scope of the literature search and evidence synthesis.

### Population (P)

Critically ill neonates (<28 days of life) requiring extracorporeal blood purification support in the setting of multiple organ dysfunction syndrome (MODS), sepsis, acute kidney injury (AKI), hyperammonemia, fluid overload, or other severe metabolic disturbances. The key terminology and abbreviations used throughout the review are summarized in Table 1.

**Table 1:** Key terminology and abbreviations used in this review.

Abbreviation	Full term
HDF	Hemodiafiltration
CKRT	Continuous Kidney Replacement Therapy
CRRT	Continuous Renal Replacement Therapy
CVVH	Continuous Veno-Venous Hemofiltration
CVVHDF	Continuous Veno-Venous Hemodiafiltration
ECMO	Extracorporeal Membrane Oxygenation
AKI	Acute Kidney Injury
MODS	Multiple Organ Dysfunction Syndrome

### Intervention (I)

Hemodiafiltration (HDF) and related continuous kidney replacement therapy (CKRT) modalities, including continuous venovenous hemodiafiltration (CVVHDF), continuous venovenous hemofiltration (CVVH), and other neonatal extracorporeal blood purification techniques.

### Comparator (C)

No formal comparator was required because this review was designed as a structured narrative review intended to provide a descriptive synthesis of published clinical experiences and observational evidence. When comparative data were available within individual studies, these findings were described narratively without formal comparative analysis.

### Primary outcome (O)

The primary outcome of interest was metabolic stabilization, including correction of acid-base disturbances and stabilization of electrolyte abnormalities following initiation of extracorporeal blood purification therapy.

### Secondary outcomes (O)

Secondary outcomes included:

- lactate reduction;
- hemodynamic stabilization;
- vasopressor requirements;
- fluid balance control;
- inflammatory biomarker trends;
- anticoagulation-related observations;
- safety outcomes and procedure-related complications;
- technical feasibility of extracorporeal support.

### Study design (S)

Observational studies, retrospective cohorts, registry reports, and case series evaluating HDF or related CKRT modalities in neonatal populations. Review articles and consensus publications were used exclusively for background information, contextual interpretation, and discussion of evolving technologies and clinical practice concepts and were not included in the descriptive synthesis of clinical outcomes.

### Scope of the review

The review focused on critically ill neonates (<28 days of life) requiring extracorporeal blood purification support in the setting of multiple organ dysfunction syndrome (MODS), sepsis, acute kidney injury (AKI), hyperammonemia, fluid overload, or other severe metabolic disturbances.

Particular attention was given to reports describing metabolic, hemodynamic, safety-related, and technical outcomes associated with neonatal extracorporeal blood purification therapies, as specified in the PICOS framework.

When comparative data were available within individual studies, these findings were described narratively. No formal comparative analysis was performed.

### Literature identification

Relevant literature was identified through searches of PubMed/MEDLINE, Scopus, Web of Science Core Collection, and the Cochrane Library.

The search focused on publications addressing neonatal hemodiafiltration, continuous kidney replacement therapy, acute kidney injury, sepsis, hyperammonemia, and extracorporeal blood purification techniques.

The search period covered January 2018 through October 2025. Additional relevant publications were identified through manual examination of reference lists from selected articles.

### Search strategy

A structured literature search was conducted in PubMed/MEDLINE, Scopus, Web of Science Core Collection, and the Cochrane Library to identify publications relevant to neonatal hemodiafiltration and related extracorporeal blood purification therapies.

The search strategy combined controlled vocabulary (where applicable) and free-text keywords using Boolean operators (AND, OR). Representative search terms included:

- “neonate” OR “newborn” OR “neonatal”;
- AND “hemodiafiltration” OR “continuous venovenous hemodiafiltration” OR “CVVHDF”;
- OR “continuous kidney replacement therapy” OR “CKRT” OR “continuous renal replacement therapy” OR “CRRT”;
- AND “acute kidney injury” OR “AKI”;
- OR “multiple organ dysfunction syndrome” OR “MODS”;
- OR “sepsis”;
- OR “hyperammonemia”;
- OR “extracorporeal blood purification”.
- representative PubMed search string was:
- (“neonate” OR “newborn” OR neonatal)

AND

- (“hemodiafiltration” OR CVVHDF OR “continuous kidney replacement therapy” OR CKRT OR CRRT)

AND

- (“acute kidney injury” OR AKI OR sepsis OR MODS OR hyperammonemia)

The search was limited to publications published between January 2018 and October 2025. Only English-language articles involving neonatal populations were considered. Additional relevant publications were identified by manually screening the reference lists of eligible studies.

### Study selection, data extraction, and quality assessment

All retrieved records were exported to Microsoft Excel, where duplicates were identified electronically and verified manually. Two reviewers independently screened titles, abstracts, and full texts according to the PICOS criteria, recorded reasons for exclusion, and extracted data using a standardized form; disagreements were resolved by consensus. Methodological quality was evaluated based on study design, sample size, completeness of outcome reporting, and potential sources of bias and was considered when interpreting the evidence.

The literature identification and study selection process is summarized in Figure 1.

**Figure 1:**
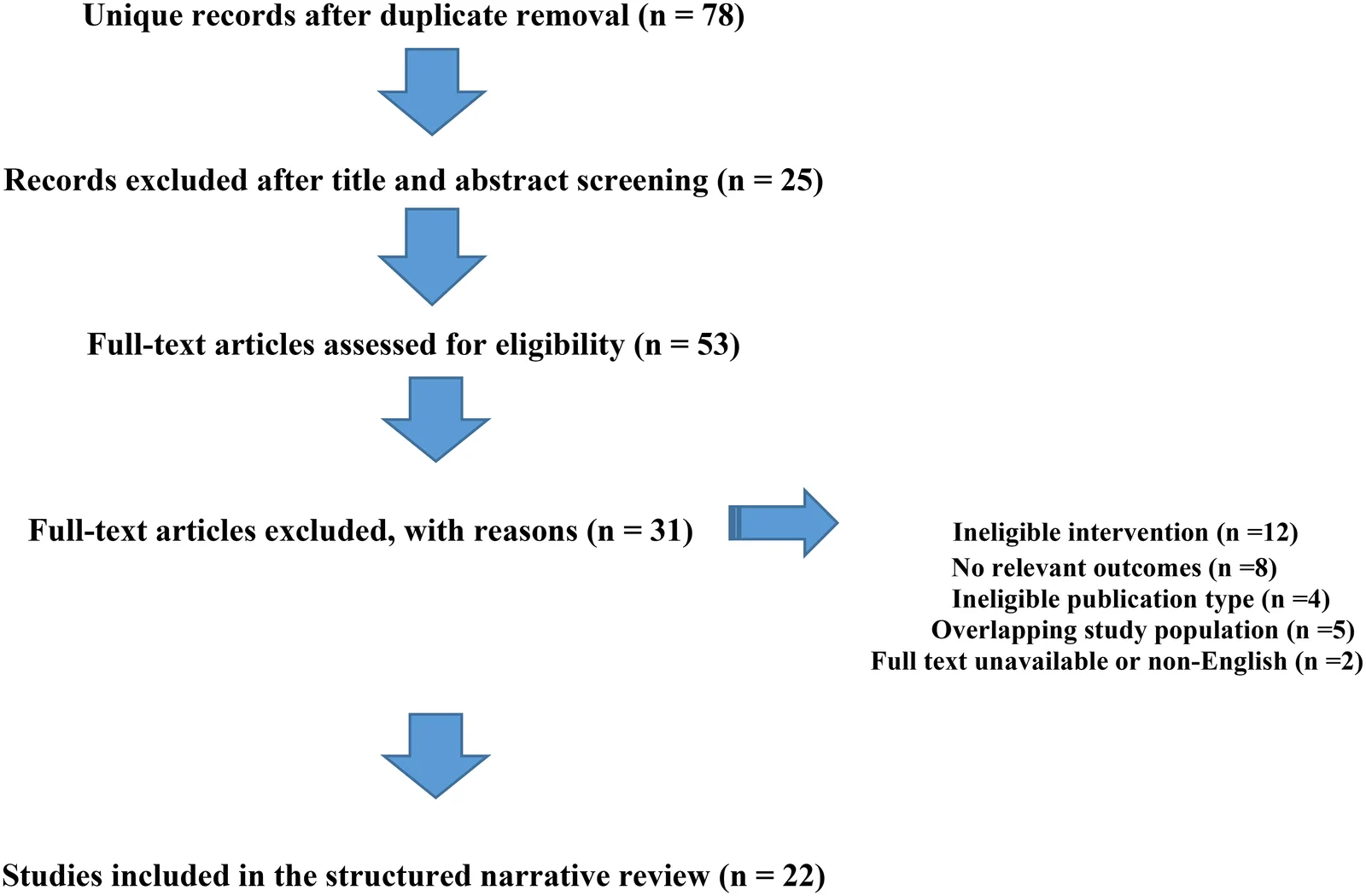
Flow diagram of literature identification and study selection.

### Data collection and narrative synthesis

Information from relevant publications was summarized descriptively, including:
- publication characteristics;
- patient populations;
- clinical indications;
- extracorporeal techniques;
- anticoagulation approaches;
- treatment characteristics;
- reported outcomes;
- complications and safety observations.
The evidence was synthesized narratively and organized according to major thematic domains:
- clinical indications;
- technical aspects of therapy;
- metabolic effects;
- hemodynamic effects;
- anticoagulation strategies;
- safety considerations;
- emerging neonatal technologies.

### Interpretation of evidence

Because of the substantial heterogeneity of the available literature, findings were interpreted descriptively.

Reported outcome ranges represent observations reported in individual studies and should not be interpreted as pooled quantitative estimates, epidemiological prevalence measures, or meta-analytic calculations.

The interpretation of findings considered important limitations of the existing evidence base, including small sample sizes, observational study designs, heterogeneous patient populations, variability in treatment protocols, and inconsistent outcome reporting.

In addition, evidence was interpreted according to the type and methodological strength of the included publications. Narrative reviews, systematic reviews, and consensus statements were used primarily to provide background context and describe current concepts in neonatal extracorporeal kidney support. Clinical conclusions regarding efficacy, safety, and patient outcomes were based predominantly on original clinical studies, including observational cohorts, registry analyses, retrospective studies, and case series. Given the predominance of non-randomized evidence, findings were interpreted cautiously with consideration of study design, sample size, potential sources of bias, and methodological limitations.

### Methodological transparency

The methodological approach was designed to maximize transparency and reproducibility while maintaining the flexibility inherent to a structured narrative review. Literature identification, study selection, and evidence synthesis were performed according to predefined methodological principles. Owing to the substantial clinical and methodological heterogeneity across the available studies, findings were synthesized descriptively rather than quantitatively.

## Results

### Characteristics of the reviewed literature

The identified publications primarily comprised retrospective cohort studies, registry-based analyses, clinical series, and observational investigations examining the application of hemodiafiltration (HDF) and other continuous kidney replacement therapy (CKRT) modalities in critically ill neonates.

The most commonly reported indications for extracorporeal kidney support were acute kidney injury (AKI), sepsis, multiple organ dysfunction syndrome (MODS), hyperammonemia, severe metabolic derangements, and fluid overload.

Among the extracorporeal platforms, CARPEDIEM™, NIDUS, Prismaflex HF20, and Aquarius systems were most frequently utilized. Therapeutic approaches included continuous venovenous hemodiafiltration (CVVHDF), continuous venovenous hemofiltration (CVVH), and other CKRT-based techniques. Anticoagulation was predominantly achieved using unfractionated heparin or regional citrate anticoagulation.

### Metabolic and hemodynamic findings

Across the reviewed studies, extracorporeal kidney support was consistently associated with improved metabolic control, including correction of acid-base imbalance, reduction of lactate levels, and stabilization of electrolyte disturbances.

Evidence regarding the hemodynamic and inflammatory effects of extracorporeal therapy remains limited. In a single neonatal case report, initiation of CVVH was followed by hemodynamic stabilization, reduced vasopressor doses, and decreases in C-reactive protein from 297 to 19 mg/L and procalcitonin from 24.7 to 1.1 ng/mL (42). However, because these findings were derived from a single patient receiving concurrent intensive care, they should be interpreted as descriptive and hypothesis-generating rather than as evidence of a causal treatment effect.

Overall, methodological quality was limited by small sample sizes, predominantly retrospective single-center designs, absent comparator groups, and heterogeneous outcome reporting. These limitations were considered in the narrative synthesis.

### Safety profile

Reported adverse events included transient hypotension, circuit thrombosis, electrolyte abnormalities, vascular access complications, and occasional bleeding episodes.

The occurrence and severity of complications differed substantially among studies and appeared to depend on patient characteristics, device type, anticoagulation strategy, and center-specific expertise.

## Clinical applications in specific neonatal populations

### Sepsis

Available clinical reports indicate that extracorporeal blood purification may facilitate metabolic stabilization and cardiovascular support in neonates with severe sepsis. Improvements in circulatory performance and reductions in vasopressor requirements were frequently noted during treatment.

### Multiple organ dysfunction syndrome

In neonates with MODS, HDF and related CKRT modalities were primarily applied as supportive interventions to maintain metabolic balance, optimize fluid status, and provide temporary organ support while definitive treatment of the underlying condition was undertaken.

### Preterm and low-birth-weight neonates

The reviewed studies demonstrated the technical feasibility of individualized low-flow extracorporeal support in preterm and extremely low-birth-weight infants. However, experience remains restricted to relatively small cohorts, and robust outcome data are still scarce.

### Combined ECMO and CKRT support

Several publications evaluated the integration of CKRT or HDF with extracorporeal membrane oxygenation (ECMO) in critically ill neonates. These reports suggested improved fluid management and enhanced metabolic control during extracorporeal support; however, the available data remain insufficient to establish definitive therapeutic advantages.

### Summary of the available evidence

Collectively, the reviewed studies suggest that HDF and related CKRT modalities may serve as valuable supportive therapies for metabolic stabilization, fluid management, and hemodynamic optimization in selected critically ill neonatal populations.

At the same time, interpretation of the available literature is constrained by the predominance of non-randomized study designs, relatively small cohorts, clinical heterogeneity, and substantial variation in treatment protocols and outcome definitions. Therefore, the current body of evidence should be regarded as exploratory and hypothesis-generating rather than conclusive proof of therapeutic superiority or effectiveness.

## Discussion

The application of hemodiafiltration (HDF) and related continuous kidney replacement therapy (CKRT) modalities in critically ill neonates has received growing attention over the past decade, particularly in the management of multiple organ dysfunction syndrome (MODS), severe sepsis, acute kidney injury (AKI), and hyperammonemia. Despite increasing clinical utilization, the current literature is characterized by relatively small cohorts, registry-based analyses, retrospective investigations, and single-center experiences.

From a physiological perspective, HDF integrates diffusive and convective clearance mechanisms, allowing the removal of both low-molecular-weight solutes and selected middle-molecular-weight substances. This combined approach has generated interest in the potential role of HDF as a supportive extracorporeal strategy in neonatal intensive care. Nevertheless, the extent to which these physiological advantages translate into measurable clinical benefit remains uncertain.

Across the reviewed studies, extracorporeal kidney support was associated with improvements in metabolic homeostasis, including correction of acid-base imbalance, normalization of electrolyte disturbances, and reduction of lactate concentrations. Several investigators also documented improved cardiovascular stability and decreased vasopressor requirements during treatment. Interpretation of these findings, however, remains challenging because of differences in patient characteristics, treatment indications, device configurations, and outcome definitions.

Fluid overload represents an additional therapeutic target in neonatal critical care. Excessive fluid accumulation has been linked to adverse outcomes, particularly in neonates with cardiac dysfunction, renal impairment, or severe systemic inflammation. Available data suggest that HDF and related CKRT techniques can facilitate individualized fluid management; however, adequately powered comparative studies are still lacking.

### Comparative interpretation of current evidence

An important question arising from the current literature is whether HDF provides clinically relevant advantages over alternative CKRT modalities, particularly continuous venovenous hemofiltration (CVVH). Theoretically, the combination of convective and diffusive clearance may enhance solute removal while maintaining efficient clearance of middle molecules. However, neonatal studies directly comparing HDF and CVVH remain scarce, preventing reliable assessment of relative efficacy.

Current data indicate that extracorporeal kidney support may be particularly valuable in neonates with severe fluid overload, sepsis-associated MODS, AKI, hyperammonemia, and profound metabolic instability. In these clinical scenarios, HDF and related techniques appear capable of supporting metabolic regulation, fluid balance, and renal function while definitive treatment of the underlying disease is pursued. Nevertheless, considerable variation in patient selection practices persists among institutions.

A consistent finding across the reviewed literature is improvement in surrogate physiological markers, including lactate clearance, electrolyte normalization, correction of acid-base disturbances, and stabilization of hemodynamic parameters. In contrast, evidence supporting a direct survival advantage remains considerably less convincing. Outcomes in critically ill neonates are strongly influenced by congenital anomalies, severity of organ dysfunction, infectious burden, and coexisting comorbidities, making attribution of survival benefit to extracorporeal therapies alone difficult.

Several unresolved questions remain. These include the lack of randomized controlled trials, scarcity of prospective multicenter investigations, absence of standardized anticoagulation approaches, variability in outcome reporting, and limited information regarding long-term neurodevelopmental outcomes. Addressing these issues should represent a priority for future collaborative research initiatives.

### Technical considerations

The implementation of extracorporeal therapies in neonates remains technically demanding. Small circulating blood volume, challenges in vascular access, anticoagulation management, and susceptibility to hemodynamic instability continue to represent major obstacles.

The introduction of neonatal-specific platforms such as CARPEDIEM™, NIDUS, and other low-volume CKRT systems has substantially improved technical feasibility in low-weight infants. Nevertheless, much of the published experience originates from highly specialized centers with extensive expertise, which may limit broader applicability.

Table 2 demonstrates that contemporary review articles consistently identify CKRT, including HDF, as a feasible supportive therapy for critically ill neonates. Across the available reviews, metabolic stabilization, fluid balance optimization, and correction of electrolyte disturbances represent the most consistently reported clinical benefits. At the same time, the evidence uniformly highlights the lack of prospective multicenter studies and standardized treatment protocols, emphasizing the need for further high-quality clinical research.

**Table 2:** Review articles and conceptual publications on continuous kidney replacement therapy in neonates and infants.

Authors	Article title	Journal	Year	Research focus	Technique/modality used	Sample size (*n*)	Gestational age	Body weight (kg)	Anticoagulation strategy	Indications	Key outcomes	Complications
Cortina G, Daverio M, Demirkol D, Chanchlani R, Deep A, et al.	Continuous renal replacement therapy in neonates and children: what does the pediatrician need to know? An overview from the Critical Care Nephrology Section of the European Society of Paediatric and Neonatal Intensive Care (ESPNIC)	Eur J Pediatr	2024	CRRT review in neonates and children	CRRT/CKRT	N/A	N/A	N/A	Heparin and citrate anticoagulation discussed	AKI, fluid overload, sepsis, intoxications, liver failure, metabolic disorders	Comprehensive review of CRRT in neonates and children; emphasizes early referral and follow-up after AKI/CRRT	Not specifically analyzed; common CRRT-related complications discussed.
Slagle C, Askenazi D, Starr M, et al.	Recent Advances in Kidney Replacement Therapy in Infants: A Review	Am J Kidney Dis	2024	Advances in KRT in infants	KRT, включая CARPEDIEM/NIDUS и др	N/A	N/A	N/A	Heparin and citrate anticoagulation discussed	AKI, fluid overload, kidney failure, inborn errors of metabolism, severe electrolyte abnormalities	Reviews recent advances in infant KRT, including CARPEDIEM and NIDUS; discusses benefits, limitations, ethical considerations, and future directions	Bleeding, thrombosis, catheter-related complications, hemodynamic instability, electrolyte disturbances discussed
Chu X, et al.	Continuous renal replacement therapy in neonates with AKI and MODS: narrative review	Italian Journal of Pediatrics	2025	CRRT review in neonates with AKI, MODS, sepsis, and hyperammonemia	CRRT/CKRT	N/A	N/A	N/A	Heparin and Regional Citrate Anticoagulation (RCA) discussed	AKI, MODS, sepsis, hyperammonemia, fluid overload	Narrative review of CRRT in neonates; discusses indications, technical considerations, anticoagulation, and neonatal-specific devices	Bleeding, thrombosis, circuit clotting, electrolyte disturbances, hemodynamic instability
Raina R, et al.	Hemodialysis in neonates and infants: A systematic review	Semin Dial	2018	State-of-the-art CRRT review in children	Hemodialysis	N/A	N/A	N/A	Heparin anticoagulation discussed	AKI, hyperammonemia, metabolic disorders, fluid overload, intoxications	Systematic review demonstrating feasibility of hemodialysis in neonates and infants; advances in vascular access and dialysis technology improved outcomes	Hypotension, vascular access complications, bleeding, thrombosis, electrolyte disturbances
Gist KM, et al.	Acute kidney injury, fluid balance, and continuous renal replacement therapy in children and neonates treated with extracorporeal membrane oxygenation	Curr Pediatr Rep	2024	Review of AKI, fluid overload and CRRT during ECMO	ECMO + CRRT	N/A	N/A	N/A	Systemic heparin anticoagulation during ECMO; CRRT anticoagulation approaches discussed	AKI, fluid overload, electrolyte disturbances during ECMO support	Reviews the impact of AKI and fluid overload on ECMO outcomes; supports early CRRT integration for fluid management and kidney support	Bleeding, thrombosis, hemolysis, circuit clotting, electrolyte disturbances, hemodynamic instability
Govindan S, et al.	Kidney replacement therapy for infants: CARPEDIEM and NIDUS to enhance precision in clearance	Kidney Int Rep	2024	Infant-specific kidney replacement therapy devices	CARPEDIEM, NIDUS	N/A	N/A	N/A	Heparin and citrate anticoagulation approaches discussed	AKI, fluid overload, hyperammonemia, metabolic disorders, neonatal kidney support	Infant-specific KRT devices (CARPEDIEM and NIDUS) improve precision and feasibility of extracorporeal support in low-weight infants	Bleeding, thrombosis, circuit clotting, vascular access complications, electrolyte disturbances

Table 2 summarizes review articles and conceptual publications that provide the current theoretical framework for neonatal CKRT, including indications, technical considerations, anticoagulation strategies, and emerging technologies.

### Controversies in clinical indications

The optimal timing and indications for HDF initiation remain incompletely defined. Although AKI, severe fluid overload, and hyperammonemia are generally accepted indications for extracorporeal kidney support, the role of HDF in neonatal sepsis and MODS remains less clearly established.

Clinical practice varies considerably among institutions. Some centers favor earlier initiation in selected high-risk patients, whereas others reserve extracorporeal therapy for refractory clinical deterioration. Such differences likely reflect variations in institutional resources, experience, and local treatment protocols.

### HDF in combination with ECMO

Integration of CKRT or HDF with extracorporeal membrane oxygenation (ECMO) represents an area of increasing clinical interest. Available reports suggest that combined extracorporeal support may improve fluid balance management and metabolic control in neonates requiring ECMO.

At present, however, available information is largely derived from small clinical cohorts and center-specific experiences. Consequently, the optimal timing, technical configuration, and anticoagulation strategies for combined ECMO-CKRT support remain to be established.

## Future perspectives

Future investigations should prioritize prospective multicenter studies, standardized reporting of clinical outcomes, and harmonization of treatment protocols. Greater consistency in patient selection criteria, anticoagulation strategies, and outcome definitions would improve comparability across studies and facilitate more reliable assessment of therapeutic effectiveness

## Conclusion of discussion

Collectively, the reviewed literature indicates that HDF may represent a feasible supportive extracorporeal intervention for selected critically ill neonates. Although encouraging effects on metabolic control, fluid management, and physiological stabilization have been reported, the current knowledge base remains insufficient to establish definitive therapeutic superiority. Reported improvements in vasopressor requirements and inflammatory biomarkers are based largely on isolated observational reports and should therefore be considered preliminary and hypothesis-generating. Therefore, available findings should be regarded as preliminary clinical evidence that supports further investigation rather than conclusive proof of efficacy.

Table 3 summarizes the currently available primary clinical evidence evaluating CKRT and HDF in neonatal populations. Most published studies are retrospective cohorts or case series originating from highly specialized centers with limited sample sizes. Despite this limitation, the findings consistently demonstrate technical feasibility, effective metabolic and fluid control, and acceptable safety profiles when therapy is performed by experienced multidisciplinary teams. Nevertheless, robust evidence regarding survival benefit and long-term outcomes remains insufficient.

**Table 3:** Primary clinical studies evaluating continuous kidney replacement therapy in neonates and young infants.

Authors	Article title	Journal	Year	Research focus	Technique/modality used	Sample size (*n*)	Gestational age	Body Weight (kg)	Anticoagulation strategy	Indications	Key outcomes	Complications	Survival
Regiroli G, Loi B, Pezza L, Sartorius V, Foti A, Barra PF, et al.	Continuous Venovenous Hemofiltration Performed by Neonatologists With Cardio-Renal Pediatric Dialysis Emergency Machine to Treat Fluid Overload During Multiple Organ Dysfunction Syndrome: A Case Series	Pediatr Crit Care Med	2023	CVVH in critically ill neonates with MODS	CVVH на CARPEDIEM	3 neonates	35-40weeks	2.6-4.1kg	Unfractionated heparin	Fluid overload, MODS, AKI, severe critical illness	Successful fluid removal, improvement of hemodynamic and metabolic status; CVVH feasible when performed by trained neonatologists	No major bleeding; occasional circuit clotting	2/3 survived (66.7%)
Battista J, et al.	CARPEDIEM® for continuous kidney replacement therapy in neonates and small infants: a French multicenter retrospective study	Pediatr Nephrol	2023	CARPEDIEM multicenter retrospective study	CARPEDIEM	41 patients	Median 39 weeks	Median 3.3 kg	Unfractionated heparin and regional citrate anticoagulation	AKI, fluid overload, metabolic disorders, hyperammonemia	CARPEDIEM was feasible and effective in neonates and small infants; good circuit performance and precise fluid management	Circuit clotting, catheter-related complications, bleeding episodes, electrolyte disturbances	Approximately 61% survival to hospital discharge
Lambert HJ, et al.	The Infant KIdney Dialysis and Utrafiltration (I-KID) Study: A Stepped-Wedge Cluster-Randomized Study in Infants Comparing Peritoneal Dialysis, Continuous Venovenous Hemofiltration, and Newcastle Infant Dialysis and Ultrafiltration System	Pediatr Crit Care Med	2023	Comparison of NIDUS with PD and CVVH in infants	NIDUS, PD, CVVH	97 infants	Term and preterm infants	0.8–7.9kg	Heparin anticoagulation for extracorporeal modalities	AKI, fluid overload, metabolic disorders, kidney failure requiring KRT	NIDUS demonstrated superior precision of fluid removal and clearance in infants <8 kg.	Circuit clotting, vascular access complications, bleeding, electrolyte disturbances	Comparable survival across treatment modalities.
Rsovac S, et al.	Continuous Renal Replacement Therapy in Critically-Ill Term and Preterm Newborns: A Single-Center Study in Belgrade	Children (Basel)	2025	Survival and prognostic factors during CRRT in critically ill neonates	CRRT	34 neonates	24–40 weeks	0.6–4.8kg	Unfractionated heparin	AKI, fluid overload, sepsis, MODS, metabolic disorders	CRRT feasible in term and preterm neonates; survival influenced by illness severity and timing of CRRT initiation.	Hypotension, thrombocytopenia, circuit clotting, electrolyte disturbances, bleeding episodes	44.1% overall survival
Otsuka H, et al.	Catheter diameter and infection control are important in neonatal continuous kidney replacement therapy: a single-center retrospective study	Ren Replace Ther	2025	Catheter diameter and infection control affecting circuit life in neonatal CKRT	CKRT	22 neonates	25–40 weeks	0.7–4.5kg	Heparin-based anticoagulation	AKI, fluid overload, sepsis, metabolic disorders	Catheter diameter and infection control significantly affected circuit lifespan and CKRT performance in neonates	Catheter-related infections, circuit clotting, bleeding, thrombosis	Survival associated with longer circuit life and lower infection rates
Deja A, et al.	Factors influencing circuit lifetime in paediatric continuous kidney replacement therapies: results from the EurAKId registry	Pediatr Nephrol	2024	Factors influencing CKRT circuit lifespan	CKRT; RCA vs heparin vs no anticoagulation	1,011patients	Not reported (mixed pediatric cohort including neonates)	Median 10.2 kg	RCA significantly prolonged circuit lifespan compared with heparin and no anticoagulation.	AKI, fluid overload, metabolic disorders	Regional citrate anticoagulation significantly prolonged CKRT circuit lifespan compared with heparin or no anticoagulation.	Circuit clotting, bleeding events, anticoagulation-related complications.	Not primary outcome; not reported separately
Vuong KT, et al.	Clearance and nutrition in neonatal continuous kidney replacement therapy using the Carpediem™ system	Pediatr Nephrol	2024	Clearance, nutrition and growth in infants on CARPEDIEM CKRT	CARPEDIEM	8 neonates	Not reported	2,870 g (2.87 kg)	regional citrate anticoagulation	Neonatal kidney failure (7/8 with KF), prolonged CKRT, need for nutritional and metabolic support	Increased nutritional delivery, improved nitrogen balance, enhanced solute clearance, weight gain after day 14, successful transition to PD	Delayed growth velocity despite adequate caloric and protein delivery	
Sun Y, et al.	Effectiveness and feasibility of continuous renal replacement therapy for acute kidney injury in neonates weighing 3 kg or less: a two-centre retrospective study	BMJ Paediatr Open	2024	Feasibility and effectiveness of CRRT in neonates ≤3 kg	CRRT	19 neonates	36.71 ± 3.20 weeks	Birth weight: 2.66 kg (IQR 2.00–2.87); Weight at CRRT initiation: 2.67 kg (2.20–2.85)	Heparin anticoagulation, initial dose 10–20 U/kg/h	AKI with haemodynamic disturbance, cerebral oedema/increased intracranial pressure, cardiac insufficiency, hypercatabolism, severe fluid overload or pulmonary oedema	Significant reduction in serum creatinine and BUN; significant increase in urine output and MAP; improvement in kidney and circulatory function	Thrombocytopenia, electrolyte disturbances, hypotension, catheter clotting, IVH.	5/19 survived (26.3%)
Battista J, et al.	Continuous kidney replacement therapy with CARPEDIEM® in neonates with a birth weight below 2.5 kg	Pediatr Nephrol	2025	CKRT/CVVH in neonates <2.5 kg	CARPEDIEM	10 neonates	Median 31 weeks (IQR 29–32; range 25–38)	Birth weight median 1.1 kg (IQR 1.0–1.7; range 0.6–2.0)	Unfractionated heparin, median 10 IU/kg/h (range 8–12 IU/kg/h)	AKI, fluid overload, multiorgan dysfunction syndrome (MODS) in preterm and low-birth-weight neonates (<2.5 kg)	Efficient blood purification in all 22 sessions; fluid overload decreased from 29% to 16% (*p* = 0.04); significant reduction in creatinine and BUN; technically feasible treatment	Thrombocytopenia requiring platelet transfusion (14 sessions), hypotensive episodes (13 sessions), circuit clotting (3 sessions)	No deaths during CARPEDIEM treatment; 1/10 neonates survived to follow-up (10%), 9/10 died later due to multiorgan failure or severe neurological injury
Kaymaz D, et al.	Evaluation of anticoagulant methods in newborns undergoing continuous renal replacement therapy: what is the optimum method?	Pediatr Nephrol	2025	RCA vs heparin anticoagulation in neonatal CRRT	CRRT	27 neonates	Median 38.6 weeks	Birth weight 3.2 kg (2.0–4.0); CRRT initiation weight 3.1 kg (1.9–4.4)	Regional Citrate Anticoagulation (RCA) vs Systemic Heparin	Hyperammonemia 67% (18/27); MSUD 26% (7/27); AKI 7% (2/27)	RCA significantly prolonged filter lifespan compared with heparin: 18 h (10–19) vs 8 h (3–24), *p* = 0.02	Bleeding 30%; catheter complications 22%; hypothermia 56%; infection 44%; hypophosphatemia 67%; hypokalemia 59%; hypomagnesemia 56%; citrate accumulation 73% in RCA group (asymptomatic)	NICU mortality 33% (9/27); RCA 20% vs Heparin 50%, *p* = 0.13 (not significant)
Huang H, et al.	Regional citrate anticoagulation for continuous renal replacement therapy in newborns	Front Pediatr	2023	Safety and efficacy of RCA-CRRT in neonates	RCA-CRRT	20 neonates	Median ∼38 weeks	∼3.0 kg	Regional citrate anticoagulation (RCA)	AKI, fluid overload, metabolic disorders, hyperammonemia	Effective metabolic control; stable ionized calcium levels; prolonged circuit survival; adequate solute clearance	Transient electrolyte disturbances; episodes of citrate accumulation were uncommon and manageable	Majority survived CRRT course; no RCA-related mortality reported
Ames EG, et al.	Multisite retrospective review of outcomes in renal replacement therapy for neonates with inborn errors of metabolism	J Pediatr	2022	RRT outcomes in neonatal hyperammonemia and IEM	including CRRT and HD	63 neonates	Median 38 weeks (IQR approximately 36–39)	Median ∼3.1 kg	Not primary study objective; varied according to RRT modality and center practice	Hyperammonemia secondary to inborn errors of metabolism (IEM)	Rapid ammonia reduction; HD achieved highest clearance rates, whereas CRRT provided better hemodynamic tolerance and continuous control	Hypotension, electrolyte abnormalities, thrombocytopenia, circuit-related complications	Survival to discharge approximately 65%–70%
Markham C, et al.	Continuous Renal Replacement Therapy for Two Neonates With Hyperammonemia	Pediatr Crit Care Med	2021	Neonatal hyperammonemia case reports	CRRT/CKRT	2 neonates	35 and 37 weeks (individual cases)	3.4–3.8 kg	Not specifically reported	Severe hyperammonemia due to inborn errors of metabolism (citrullinemia, methylmalonic acidemia)	Rapid ammonia reduction; CRRT more effective than scavenger therapy alone for ammonia clearance	Significant complications not emphasized	1 survivor/1 death (outcome depended on underlying disease severity)
Zhang X, et al.	Optimal stage of initiating continuous renal replacement therapy in the treatment of neonatal acute kidney injury	BMC Nephrol	2022	Timing of CRRT initiation in neonatal AKI	CRRT	*n* = 25 neonates with AKI	33 + 4 to 40 + 1 weeks	2,250–4,000 g (2.25–4.0 kg)	Heparin anticoagulation during CRRT	Neonatal AKI (severe asphyxia, sepsis, congenital metabolic diseases)	Significant reduction in creatinine, urea and potassium within 24 h; increased urine output; better outcomes when CRRT initiated at AKI stage 0–1 rather than stage 2–3.	Thrombocytopenia (4), circuit obstruction (2), hypotension (2); no bleeding, thrombosis, hypothermia, or infection.	16/25 discharged (64%); 9/25 died or had irreversible neurologic injury/MODS despite renal recovery.
Cai C, et al.	Clinical effect and safety of continuous renal replacement therapy in the treatment of neonatal sepsis-related acute kidney injury	BMC Nephrol	2020	CRRT efficacy in neonatal sepsis-related AKI	CRRT	12 neonates	36.5 ± 2.8 weeks	2.85 ± 0.62 kg	Heparin	Sepsis-related AKI	Significant decrease in serum creatinine, BUN, potassium, inflammatory markers; improved urine output and acid-base balance	Thrombocytopenia, hypotension, catheter-related complications; no severe bleeding reported	8/12 survived (66.7%)
Mok TYD, Tseng MH, Chiang MC, et al.	Renal replacement therapy in the neonatal intensive care unit	Pediatr Neonatol	2018	RRT review in NICU	PD, HD, CRRT/RRT	17 neonates	Median 37 weeks (32–39)	Median 2.7 kg (1.5–3.6)	Heparin for (CRRT)	AKI, inborn errors of metabolism (IEM), fluid overload, metabolic derangements	CRRT effective and feasible even in low-birth-weight neonates; ammoniareduction 87.2% in IEM patients; higher efficacy than PD	Hypokalemia, hypocalcemia, hypophosphatemia, hypotension, catheter-related complications	All IEM neonates survived; mortality in non-IEM neonates was 78% (CRRT) and 80% (PD)

Table 3 summarizes primary clinical studies evaluating CKRT in neonates and young infants. These studies provide evidence regarding the feasibility, safety, anticoagulation strategies, technical performance, and clinical outcomes of extracorporeal kidney support across diverse neonatal conditions, including acute kidney injury, sepsis, hyperammonemia, and multiple organ dysfunction syndrome.

## Limitations

This structured narrative review has several limitations that should be considered when interpreting the findings.

First, the available literature consists largely of retrospective investigations, registry-based analyses, and clinical case series. The absence of randomized controlled trials and the relatively small cohorts included in many studies reduce the overall strength of the evidence and limit the generalizability of reported findings.

Second, substantial variability exists among studies regarding patient characteristics, underlying diagnoses, indications for therapy, extracorporeal platforms, anticoagulation approaches, treatment protocols, and outcome measures. Such differences complicate direct comparisons and hinder consistent interpretation of reported results.

Third, publication bias cannot be excluded, as studies reporting favorable clinical experiences may be more likely to reach publication than those describing neutral or negative outcomes.

Additionally, reporting of clinical, laboratory, and long-term outcomes was frequently inconsistent, limiting assessment of both efficacy and safety across studies.

Finally, this work was conducted as a structured narrative review incorporating systematic search elements. Consequently, no formal meta-analysis, pooled effect estimates, or comparative effectiveness analyses were performed.

### Clinical implications and future perspectives

The available literature indicates that HDF and related CKRT modalities may represent feasible extracorporeal support strategies in selected critically ill neonates requiring metabolic, fluid, or renal support.

Published studies have described improvements in metabolic homeostasis, acid-base balance, fluid management, and physiological stabilization following initiation of extracorporeal kidney support. However, current data do not permit definitive conclusions regarding superiority over alternative CKRT modalities.

Recent technological advances, including neonatal-adapted platforms such as CARPEDIEM™, NIDUS, and modified low-volume CKRT systems, have expanded the feasibility of extracorporeal therapies in very-low-birth-weight infants and other vulnerable neonatal populations.

Despite these developments, important knowledge gaps remain, highlighting the need for prospective multicenter studies, standardized treatment protocols, and long-term outcome evaluation.

Based on the currently available evidence, HDF appears to be most beneficial for critically ill neonates with acute kidney injury (AKI), severe fluid overload, hyperammonemia, sepsis-associated multiple organ dysfunction syndrome (MODS), and profound metabolic instability that cannot be adequately corrected by conventional intensive care alone. In these clinical scenarios, HDF may facilitate metabolic stabilization, optimize fluid balance, and provide temporary organ support while definitive treatment of the underlying disease is implemented.

However, the current evidence remains limited regarding the optimal timing of HDF initiation, patient selection criteria, standardized anticoagulation protocols, comparative effectiveness versus other CKRT modalities, and long-term neurodevelopmental outcomes. Therefore, treatment decisions should remain individualized and guided by multidisciplinary clinical expertise until stronger prospective evidence becomes available.

The decision to initiate HDF or other extracorporeal kidney support modalities in critically ill neonates should always be made by a multidisciplinary team. Careful assessment by neonatologists, pediatric intensivists, nephrologists, anesthesiologists, cardiac surgeons (when applicable), and specialized nursing staff is essential to evaluate the potential benefits, procedural risks, technical feasibility, and overall therapeutic goals for each individual patient.

Importantly, extracorporeal kidney support should not be considered a standalone treatment for neonatal sepsis or multiple organ dysfunction syndrome. Rather, it should be viewed as an adjunctive supportive therapy within a comprehensive intensive care strategy that includes source control of infection, appropriate antimicrobial therapy, hemodynamic optimization, respiratory support, and correction of metabolic disturbances. When applied to carefully selected patients, HDF may provide an important therapeutic opportunity to support organ recovery while definitive treatment of the underlying disease takes effect.

## What this review adds

- Provides a structured narrative synthesis of contemporary literature addressing HDF and related CKRT modalities in neonatal intensive care.
- Summarizes current indications, technical considerations, and safety challenges associated with extracorporeal kidney support in neonates.
- Highlights the expanding role of neonatal-specific extracorporeal platforms, including CARPEDIEM™ and NIDUS, in facilitating treatment of low-weight infants.
- Identifies key knowledge gaps, including the lack of prospective multicenter investigations, standardized treatment protocols, and long-term outcome data.
- Emphasizes the need for cautious interpretation of currently available findings and the importance of future collaborative research initiatives.

## Data Availability

The original contributions presented in the study are included in the article/Supplementary Material, further inquiries can be directed to the corresponding authors.

## References

[1] Jetton JG Boohaker LJ Sethi SK Wazir S Rohatgi S Soranno DE. Incidence and outcomes of neonatal acute kidney injury (AWAKEN): a multicentre multinational cohort study. Lancet Child Adolesc Health. (2017) 1:184–94. 10.1016/S2352-4642(17)30069-X 29732396 PMC5933049

[2] Tandukar S Palevsky PM. Continuous renal replacement therapy: who, when, why, and how. Chest. (2019) 155:626–38. 10.1016/j.chest.2018.09.004 30266628 PMC6435902

[3] Lee ST Cho H. Fluid overload and outcomes in neonates receiving continuous renal replacement therapy. Pediatr Nephrol. (2016) 31:2145–52. 10.1007/s00467-016-3363-z 26975386

[4] Garzotto F Vidal E Ricci Z Paglialonga F Giordano M Laforgia N. Continuous kidney replacement therapy in critically ill neonates and infants: a retrospective analysis with a dedicated device. Pediatr Nephrol. (2020) 35:1699–705. 10.1007/s00467-020-04562-y 32440948

[5] Peruzzi L Bonaudo R Amore A Chiale F Donadio ME Vergano L. Neonatal sepsis with multi-organ failure treated with a new dialysis device specifically designed for newborns. Case Rep Nephrol Urol. (2014) 4:113–9. 10.1159/000363691 25028585 PMC4086034

[6] Menon S Broderick J Munshi R Dill L DePaoli B Fathallah-Shaykh S. Kidney support in children using an ultrafiltration device: a multicenter retrospective study. Clin J Am Soc Nephrol. (2019) 14:1432–40. 10.2215/CJN.03240319 31462396 PMC6777586

[7] Cortina G Daverio M Demirkol D Chanchlani R Deep A. Continuous renal replacement therapy in neonates and children. Pediatr Nephrol. (2024) 183:529–41. 10.1007/s00431-023-05318-0 PMC10912166 37975941

[8] Diane Mok TY Tseng M-H Chiang M-C Lin J-L Chu SM Hsu J-F. Renal replacement therapy in the neonatal intensive care unit. Pediatr Neonatol. (2018) 59:474–80. 10.1016/j.pedneo.2017.11.015 29396136

[9] Slagle C Askenazi D Starr M. Recent advances in kidney replacement therapy in infants. Am J Kidney Dis. (2024) 83:519–30. 10.1053/j.ajkd.2023.10.012 38147895

[10] Regiroli G Loi B Pezza L Sartorius V Foti A Barra PF. Continuous venovenous hemofiltration in critically ill neonates with MODS. Pediatr Crit Care Med. (2023) 24:201. 10.1097/PCC.0000000000003177 36728157

[11] Chu X Xu J Sun Y Gong X Chen D Cai C. Continuous renal replacement therapy in neonates with AKI and MODS: narrative review. Int J Pediatr. (2025) 51:275. 10.1186/s13052-025-02123-3 PMC12482357 41023706

[12] Raina R Vijayaraghavan P Kapur G Sethi SK Krishnappa V Kumar D. Hemodialysis in neonates and infants: a systematic review. Semin Dial. (2018) 31:289–99. 10.1111/sdi.12657 29105839

[13] Totapally A Bridges BC Selewski DT Zivick EE. Role of CRRT in neonatal and pediatric ECMO. Pediatr Nephrol. (2024) 32:151332. 10.1016/j.sempedsurg.2023.151332 37871460

[14] Lambert H Hiu S Coulthard MG Matthews JNS Holstein E-M Crosier J. The I-KID study using the NIDUS device. Pediatr Crit Care Med. (2023) 24(7):604–613. 10.1097/PCC.0000000000003220 36892305 PMC10317301

[15] Coulthard MG Crosier J Griffiths C Smith J Drinnan M Whitaker M. Haemodialysing babies under 8 kg with NIDUS. Arch Dis Child Fetal Neonatal Ed. (2014) 29:1873–81. 10.1007/s00467-014-2923-3 PMC4167433 25125229

[16] Munshi R Lee-Son K Hackbarth RM Quigley R Sutherland SM Echeverri J. Clinical evaluation of prismaflex HF20 in small children. Pediatr Crit Care Med. (2020) 35:2345–235. 10.1007/s00467-020-04664-7 32607769

[17] Crosier J Colosimo D Hansen R Lambert HJ Coulthard MG Ricci Z. Ultrafiltration precision in infant devices. Kidney Int Rep. (2025) 40:3549–54. 10.1007/s00467-025-06788-0 PMC12484341 40627188

[18] Bottari G Buccione E Bayrakci B Briassoulis G Carter MJ Demirkol D. Consensus on extracorporeal blood purification in pediatrics. JAMA Netw Open. (2025) 8:e2457657. 10.1001/jamanetworkopen.2024.57657 39899300

[19] Yang Y Wang X Lu X Zhang X Huang J Xiao Z. Efficacy and safety of ECMO+CRRT in children. Front Pediatr. (2025) 13. 10.3389/fped.2025.1556642 PMC11960717 40171174

[20] Short K Hanick J Bickert P Potts J Askenazi D. Growth in neonates with congenital kidney failure on CKRT. Pediatr Nephrol. (2025) 40(12):3733–3741. 10.1007/s00467-025-06887-y 40775407 PMC12549734

[21] Otsuka H Kibe M Ishihara C Tokuhisa T. Catheter size infection control and circuit life in CKRT. Ren Replace Ther. (2025) 11:15. 10.1186/s41100-025-00609-8

[22] Deja A Guzzo I Cappoli A Labbadia R Karabay Bayazit A Yildizdas D. Factors influencing circuit lifetime in paediatric continuous kidney replacement therapies — results from the EurAKId registry. Pediatr Nephrol. (2024) 39(11):3353–3362. 10.1007/s00467-024-06459-6 39023538 PMC11413113

[23] Vuong KT Vega MR Casey L Swartz SJ Srivaths P Osborne SW. Clearance and nutrition during CKRT with CARPEDIEM. Pediatr Nephrol. (2024) 39:1937–50. 10.1007/s00467-023-06237-w 38231233

[24] Sun Y Xu J Chu X Zhang W Gong X Chen D. CRRT in neonates <= 3 kg: feasibility and effectiveness. BMJ Paediatr Open. (2024) 8. 10.1136/bmjpo-2023-002241 PMC11381672 39244221

[25] Gist KM Bastero P Ricci Z Kaddourah A Strong A Chanchlani R. Acute kidney injury, fluid imbalance, and continuous renal replacement therapy in children and neonates treated with extracorporeal membrane oxygenation. Intensive Care Med – Paediatr Neonatal. (2024) 2:28. 10.1007/s44253-024-00051-4

[26] Bottari G Ranieri VM Ince C Pesenti A Aucella F Maria A. Use of extracorporeal blood purification therapies in sepsis: the current paradigm, available evidence, and future perspectives. Crit Care*.* (2024) 28(1):432. 10.1186/s13054-024-05220-7 PMC11670469 39722012

[27] Pokorná P Michaličková D Tibboel D Berner J. Meropenem pharmacokinetics in neonates on ECMO. Antibiotics (Basel). (2024) 13:419. 10.3390/antibiotics13050419 PMC11117356 38786147

[28] Govindan S Gillen MC Reidy KJ Nada A Askenazi DJ Raina R. Kidney replacement therapy for infants: CARPEDIEM/NIDUS. Kidney Int Rep. (2024) 5:3. 10.34067/KID.0000000000000484

[29] Battista J dit Trolli SE Ranchin B Bacchetta J Baleine JF De Luca D. Continuous kidney replacement therapy with CARPEDIEM® of premature and low birth weight neonates from the French registry. Pediatr Nephrol*.* (2026) 41(6):1829–1837. 10.1007/s00467-025-06896-x 40668378

[30] Kaymaz D Bayhan SA Kostekci YE Demirtas F Aslan AD Havan M. Evaluation of anticoagulant methods in newborns undergoing continuous renal replacement therapy: what is the optimum method? Eur J Pediatr*.* (2025) 184(8):473. 10.1007/s00431-025-06314-2 40632271

[31] Negishi S Girsch JH Siegler EL Bezerra ED Miyao K Sakemura RL. Regional citrate anticoagulation in neonates on CRRT. Front Pediatr. (2023) 11. 10.3389/fped.2023.1305657 38283399 PMC10811220

[32] Brachio SS Reichman V. Outcomes of neonatal RRT for hyperammonemia: multi-site. J Pediatr. (2022) 247:14–6. 10.1016/j.jpeds.2022.05.025 35577117

[33] Wu C-Y Lin Y-C Chen C-C. CRRT in low-birth-weight neonates: taiwan experience. Front Pediatr. (2021) 9. 10.3389/fped.2021.769220

[34] Markham C. Neonatal hyperammonemia treated with CKRT. Pediatr Crit Care Med. (2021) 22:9. 10.1097/PCC.0000000000002640

[35] Zhang X Hong W Li N Gong X Cai C. Optimal timing to initiate CRRT in neonatal AKI. Exp Ther Med. (2022) 24:733. 10.3892/etm.2022.11669 36382094 PMC9641160

[36] Cai C Qiu G Hong W Shen Y Gong X. Clinical effect and safety of continuous renal replacement therapy in the treatment of neonatal sepsis-related acute kidney injury. BMC Nephrol*.* (2020) 21(1):289. 10.1186/s12882-020-01945-z 32682407 PMC7368639

[37] Buccione E Guzzi F Colosimo D Tedesco B Romagnoli S Ricci Z. Continuous renal replacement therapy in critically ill children in the pediatric intensive care unit: a retrospective analysis of real-life prescriptions, complications, and outcomes. Front Pediatr. (2021) 9:669798. 10.3389/fped.2021.669798 PMC8236631 34195164

[38] Selewski DT Cornell TT Blatt NB. Fluid overload and outcomes in critically ill children requiring CRRT. Crit Care Med. (2020). 10.1097/PCC.0000000000001349

[39] Sethi SK Bunchman T Chakraborty R Raina R. Pediatric acute kidney injury: new advances in the last decade. Kidney Res Clin Pract*.* (2021) 40(1):40–51. 33663033 10.23876/j.krcp.20.074 PMC8041642

[40] Samoni S Husain-Syed F Villa G Ronco C. Continuous renal replacement therapy in critically ill patients: evolving concepts. Nat Rev Nephrol. (2021) 11:172. 10.3390/jcm11010172 PMC8745413 35011913

[41] Garzotto F Ostermann M Martín-Langerwerf D Sánchez-Sánchez M Teng J Robert R. The Dose Response Multicentre Investigation on Fluid Assessment (DoReMIFA) in critically ill patients. Critical Care*.* (2016) 20(1):196. 27334608 10.1186/s13054-016-1355-9 PMC4918119

[42] Ibrayev T Zhauarova G Baesheva D. Neonatal sepsis and the use of extracorporeal therapy methods: a clinical case. J Biol Res. (2025) 1(4):27–35. 10.70264/jbr.v1.4.2025.4

